# The new robotic platform Hugo™ RAS for lateral transabdominal adrenalectomy: a first world report of a series of five cases

**DOI:** 10.1007/s13304-022-01410-6

**Published:** 2022-11-04

**Authors:** Marco Raffaelli, Pierpaolo Gallucci, Nikolaos Voloudakis, Francesco Pennestrì, Roberto De Cicco, Giovanni Arcuri, Carmela De Crea, Rocco Bellantone

**Affiliations:** 1 U.O. Chrirurgia Endocrina e Metabolica, Fondazione Policlinico Universitario Agostino Gemelli IRCCS, Largo A. Gemelli 8, 00168 Rome, Italy; 2 Centro di Ricerca in Chirurgia delle Ghiandole Endocrine e dell’Obesità, Università Cattolica del Sacro Cuore, Rome, Italy; 3 U.O Anestesie delle Chirurgie Generali e dei Trapianti, Fondazione Policlinico Universitario Agostino Gemelli IRCCS, Rome, Italy; 4 U.O. Direzione Tecnica e Innovazione - Tecnologia Sanitaria, Fondazione Policlinico Agostino Gemelli IRCCS, Rome, Italy

**Keywords:** Hugo™ RAS, Lateral transabdominal adrenalectomy, Robotic surgery, Minimal invasive surgery, Docking

## Abstract

Robotic assisted surgery is the most rapidly developing field of minimally invasive surgery. Its wide diffusion has led to the development and standardization of robotic-assisted approaches also for adrenalectomy. In this study, we present the first five robotic-assisted lateral transabdominal adrenalectomies performed with the new Hugo RAS™ system (Medtronic, Minneapolis, MN, USA). After an official training course of the surgical team, five consecutive patients scheduled for unilateral adrenalectomy, underwent robotic-assisted operations in our institution. Patients that were candidates for partial adrenalectomy were excluded. A description of the operating theatre, robotic arms and docking setup is provided. Four female and one male patient underwent lateral transabdominal adrenalectomy, three for lesions on the left side and two on the right. Median lesion size was 3.9 cm (range: 30–90) and preoperative diagnosis was Cushing’s syndrome in three patients, an adrenal cystic lesion and a pheochromocytoma. The median docking time was 5 min (range: 5–8) and the median console time was 55 min (range: 29–108). Procedures were performed without intraoperative complications and no conversions or additional ports were needed. System’s function and docking were uneventful. Based on our initial experience, adrenalectomy with the Hugo™ system is feasible. This study provides technical notes for other centres that wish to perform robotic-assisted adrenalectomies with the Hugo™ RAS as well as general information and our preliminary insights on this new platform.

## Introduction

The first cases of robotic-assisted adrenalectomy (RAA), with the ZEUS AESOP platform (Computer Motion, Inc., Santa Barbara, CA) were reported in 1999 by Piazza et al. [1] and Hubens et al. [2]. Since then, and with the diffusion of the Da Vinci system (Intuitive Surgical, Sunnyvale, CA, USA), the feasibility and safety of RAA with the Da Vinci system has been repeatedly proven by several authors [3–14], and the results compared to other approaches in recent meta-analyses [15, 16]. The perceived advantages of robotic technology over laparoscopic adrenalectomy (LA) include improved ergonomics, stereoscopic vision, hand tremor filtration, and greater range of motion within the operative field. The above can potentially result in improved surgical dexterity and maximize surgical efficiency compared with conventional laparoscopic surgery. Those advantages have been consolidated by centers that performed challenging operations, previously thought impossible laparoscopically, via a robotic-assisted approach, such as pancreas transplantation and selective distal splenorenal shunt [17–19].

Recent promising publications and meta-analyses portray advantages of RAA with the Da Vinci systems over LA concerning hospital stay [13, 15, 16, 20] blood loss [16, 20] and complications [13], especially in particular circumstances, including obese patients (body mass index > 30 kg/m2), large, and functioning tumours (particularly pheochromocytoma) [10, 12, 14, 21, 22].

In spite of such advantages, the transition from the actual gold standard, LA, to RAA has been limited. A recent European study, including 1.005 patients from 46 centers, showed that only 18.8% of minimally invasive adrenalectomies were robotically assisted [13].

Technical aspects, limited platform availability and cost-related issues are the principal causes of the still limited diffusion of RAA. In technical matters, in the Da Vinci systems, the lack of haptic feedback remains a concern. The whole setup can also be cumbersome at times, while the docking/undocking processes are still fairly time-consuming, although they can be significantly reduced with experience and standardization of the procedure(s). The single operation cart is also bulky and takes up a lot of vital space, thus limiting access to the patient [23].

However, most of the concerns are related to increased costs with respect to LA. As early as in 2006, Winter et al. calculated that there was a need of 500 robotic operations per year performed in order for the procedure to become cost-effective [24]. In another publication by Brunaud et al. [25], the authors demonstrated that the cost of RAA was 2.3 times higher than the LA. Conversely, a recent paper from our group has demonstrated that RAA is economically sustainable in a Health Care System where inpatient care reimbursement is based on Diagnosis-Related Groups (DRGs), despite the positive margin (meaning the reimbursement minus the total cost of the operation) being significantly reduced with respect to LA [26].

The initial equipment cost of the Da Vinci systems, as well as maintenance and consumables costs, are still steep. This, in theory, can be partially attributed to the lack of a competitive environment. In addition, insurance agencies, in most countries, do not provide extra reimbursement over more common minimally invasive techniques, such as pure laparoscopy.

Several platforms for robotic-assisted surgery (RAS) are now emerging, promising improved technical specifications, and competing for a place in the marketplace, which might mitigate the obstacles of increased cost and accessibility.

Among new platforms for RAS, one of the most recently introduced systems is the Hugo™ RAS (Medtronic, Minneapolis, MN, USA). It consists of a system tower, an open console and four arm carts. Each arm moves independently, allowing various placements and reducing the risk of collision. In addition, each arm has six different joints, promising a wider manoeuvre range. The surgeon is seated on an open console, consisting of a 32-inch-widescreen HD-3D display with dedicated glasses, two innovative arm-controllers with handgrips simulating a “pistol grip” and a footswitch panel to control the camera, energy sources, and the reserve arm. A Karl Storz 3D Tipcam S™ (Karl Storz SE & Co. KG, Tuttlingen, Germany) encased in a robotic adaptor provides endoscopic vision.

The Hugo™ RAS has been introduced in the European market in March 2022, having thus far received CE (Conformité Européenne) approval for gynaecological and urological procedures, including adrenalectomy. In the United States, the Hugo™ system is still considered an investigational device and has not yet received FDA approval.

So far, the feasibility of the Hugo™ platform has already been tested in other urological procedures [27, 28] and gynaecological scenarios [29, 30] but not in adrenalectomy. The aim of this report was to assess the feasibility and provide technical details of the setup for lateral transabdominal adrenalectomy, describing the first case series. In addition, general information and our preliminary insights on this new platform are included so that other interested centers may introduce this new robotic system in their armamentarium.

## Methods

In July 2022, five consecutive informed patients underwent lateral transabdominal adrenalectomy with the Hugo™ RAS system in our Institution, a tertiary referral center for endocrine surgery. Exclusion criteria consisted only of patients that were candidates for partial adrenalectomy. No other specific criteria were used for patient selection, apart from being scheduled for minimal invasive adrenalectomy. Operations were performed by a surgeon experienced in both laparoscopic and robotic adrenalectomy (M.R.). All participating surgeons and nurses completed the technical training on HUGO™ RAS System delivered by Medtronic at the ORSI Academy, Aalst, Belgium. Informed consent of all participating patients was acquired.

### Patient position and trocar placement

After general anaesthesia, patients were positioned in a full lateral left or right decubitus for the right and left adrenalectomy, respectively. The breakpoint of the operating table was at the level of the 10th rib. A cushion was placed under the opposite flank with respect to the side of adrenalectomy. The table was flexed to maximize exposure of the space between the costal margin and the iliac crest. The right or left arm was elevated and secured, while the legs were flexed.

**Fig. 1:**
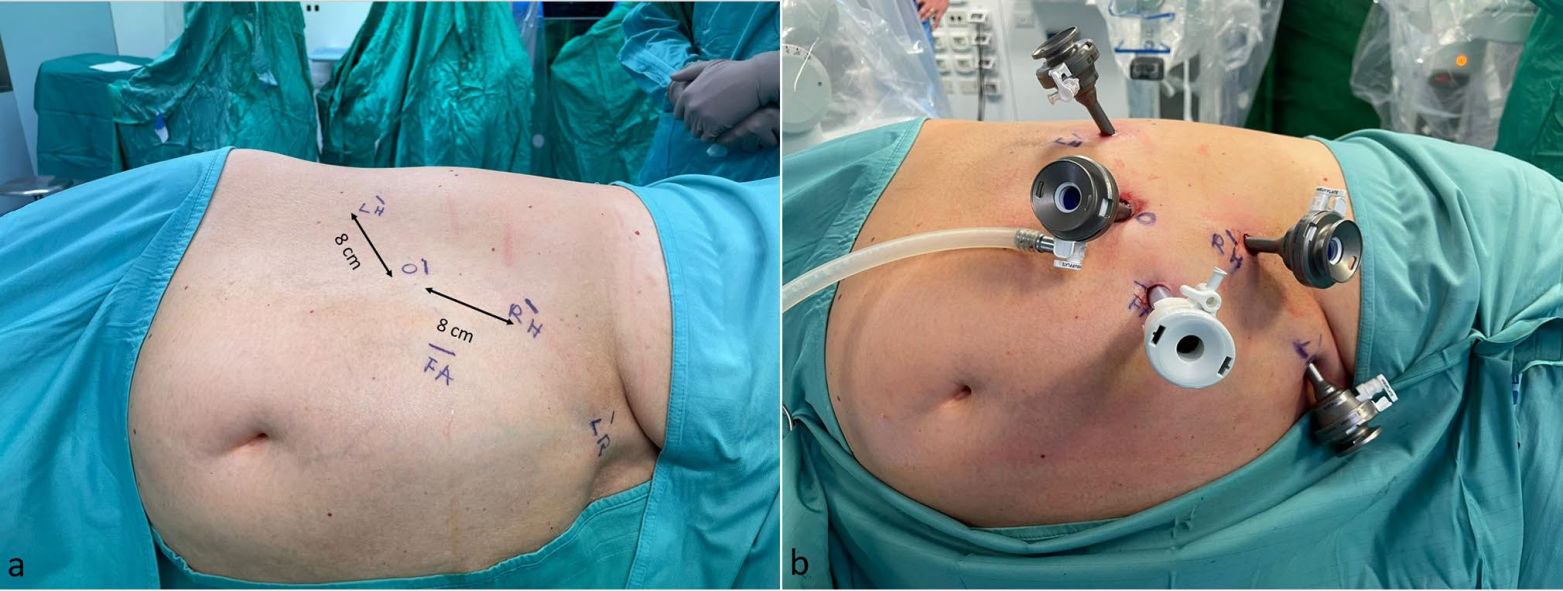
**a** Trocars position in the right lateral transabdominal Hugo™ RAS-assisted adrenalectomy. *O* endoscope, *RH* surgeon’s right hand, *LH* surgeon’s left hand, *LR* liver retractor, *FA* first assistant. **b** Trocar placement

Caution is advised when placing the robotic ports. A minimum distance of 8 cm between them is required to avoid collisions during the operation. For right adrenalectomies (Fig. 1), the first port, a 11-mm camera port, was placed along the line between the umbilicus and the right costal margin. Two 8-mm robotic trocars were then inserted medially and laterally to the first trocar (for the first operator) at about 2–3 cm below the costal margin. A 12-mm accessory trocar (fourth trocar) was placed medially, between the camera trocar and the surgeon’s right-hand trocar for the first assistant. A 5-mm trocar was positioned in the epigastrium to hold the liver retractor (2nd assistant). It still has not been tested by our team if the 4th robotic arm can substitute the 2nd assistant, since a very steep angle is necessary.

**Fig. 2:**
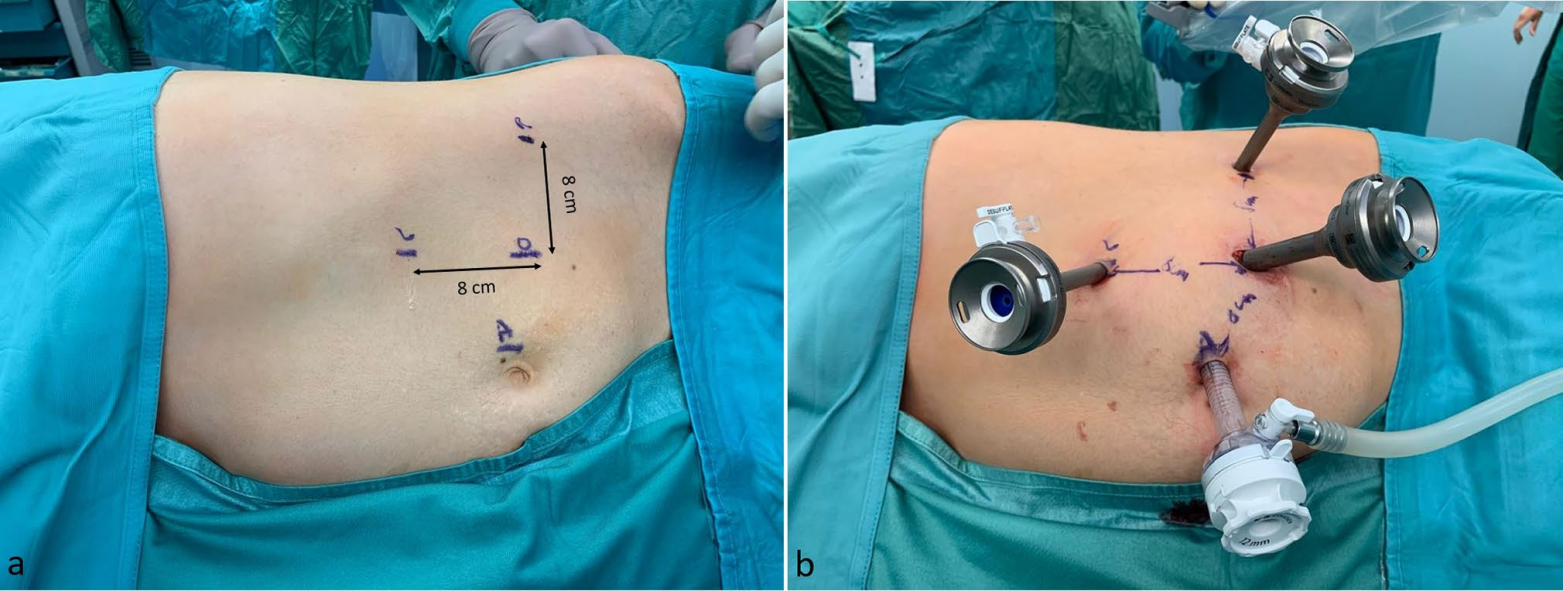
**a** Trocar position in the left lateral transabdominal Hugo™ RAS-assisted adrenalectomy. *O* endoscope, *R* surgeon’s right hand, *L* surgeon’s: left hand, *A* first assistant. **b** Trocar placement

For left adrenalectomies (Fig. 2), the 11-mm camera port was placed in the midway between the umbilicus and the left subcostal angle. The two 8-mm robotic trocars were then inserted medially and laterally to the camera port for the robotic instruments. A 12-mm accessory trocar (the fourth port) was placed medially, between the camera port and the lateral robotic port. In general, the trocars’ positions are similar to those used in the Da Vinci-assisted adrenalectomies [31].

### Docking

**Fig. 3:**
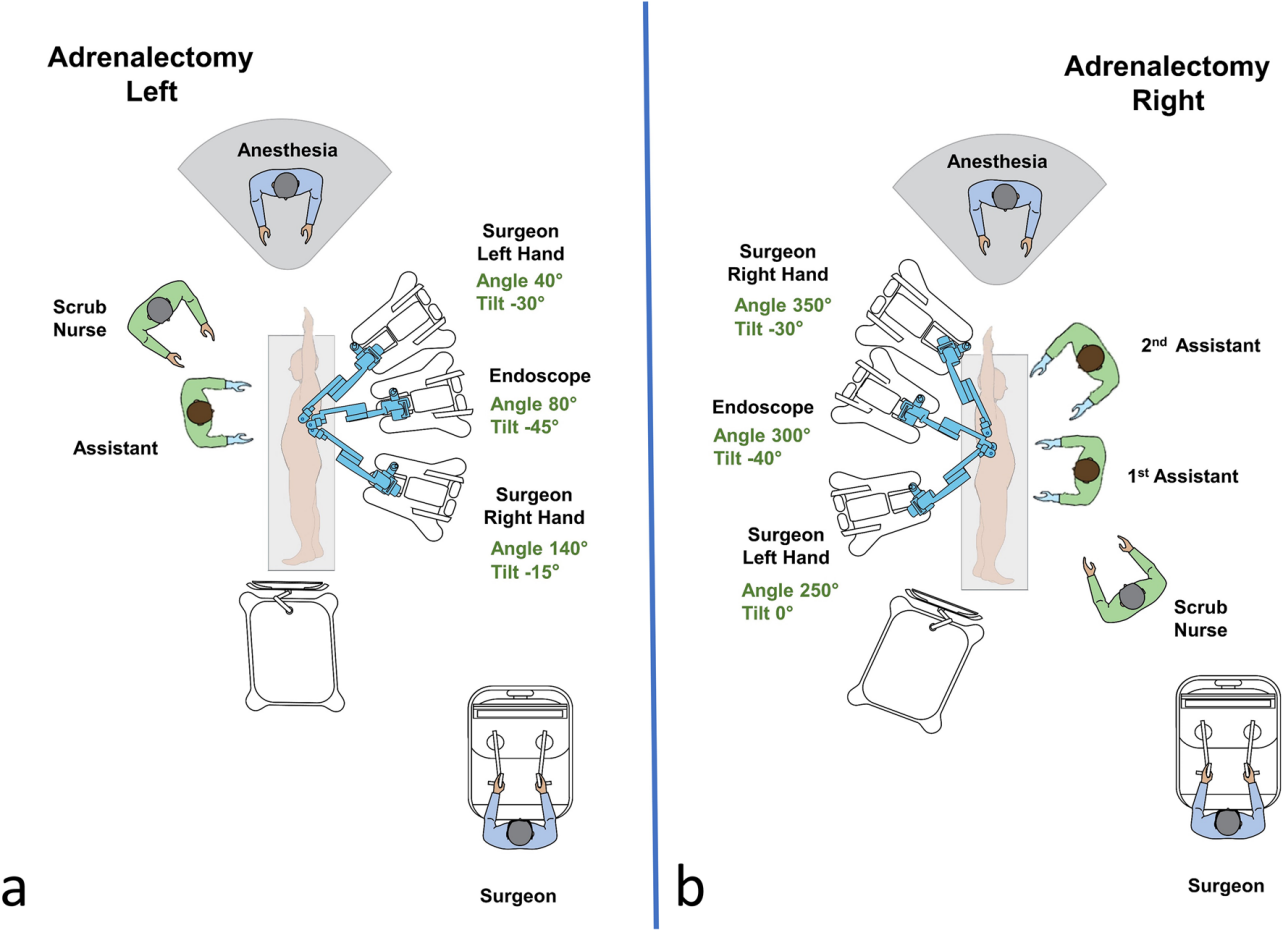
**a** Operative room settings, positions of platform’s components and surgical team members during left lateral transabdominal adrenalectomy. Description of arms docking and tilt angles. **b** Operative room setting and positions of Hugo™ RAS system and surgical team members during right lateral transabdominal adrenalectomy. Description of arms docking and tilt angles

**Fig. 4:**
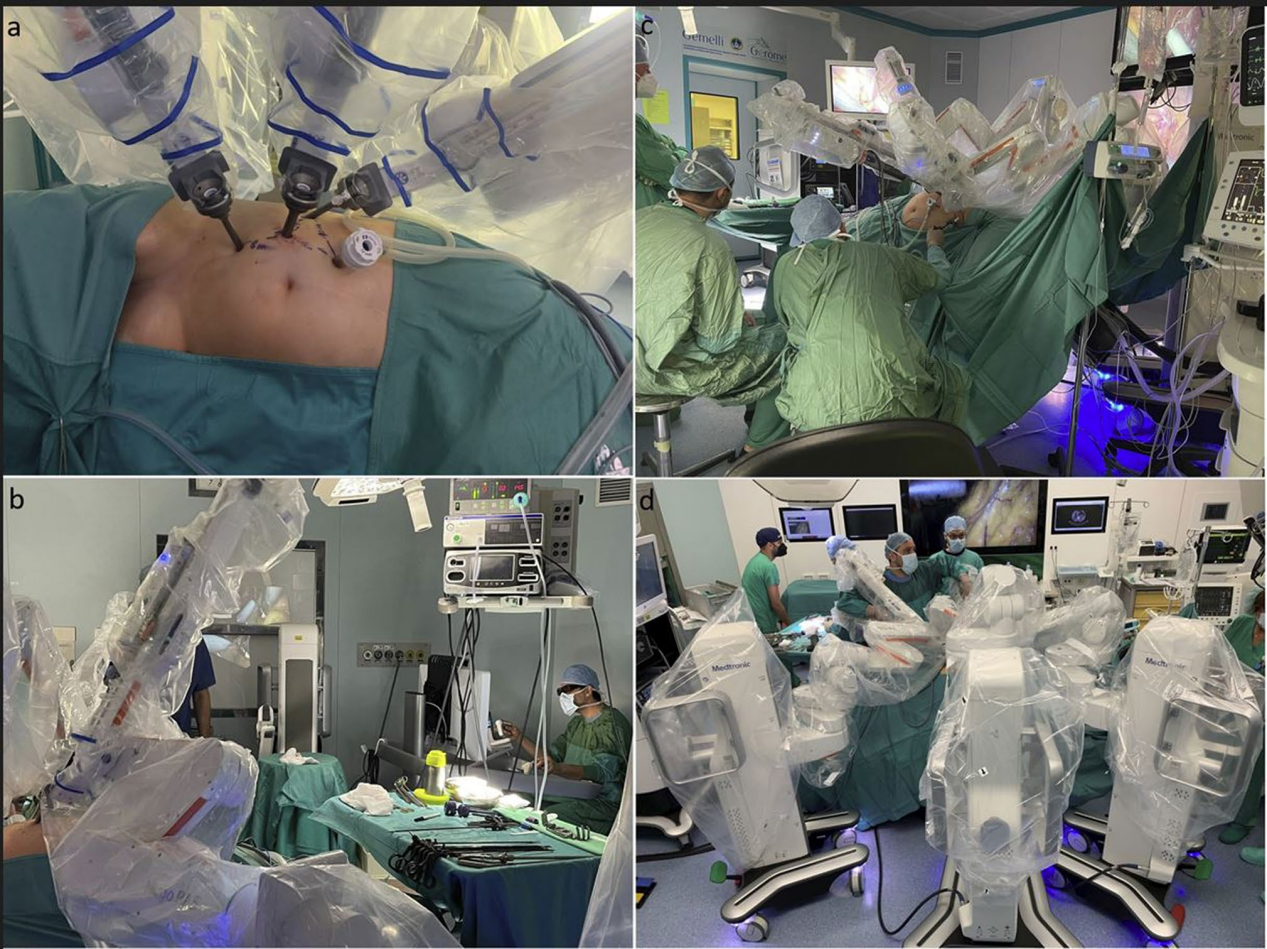
**a** Hugo™ RAS arms’ after docking in the left lateral transabdominal robotic-assisted adrenalectomy. **b** Assistants’ positions in the right lateral transabdominal robotic-assisted adrenalectomy. **c** Surgeon’s position on the Hugo™ RAS console. **d** Posterior view during a right lateral transabdominal robotic-assisted adrenalectomy

The Hugo™ system consists of 4 independent arm carts, of which three were used in adrenalectomies. Each arm requires its own settings, that can be adjusted depending on the patient’s body type. Two main settings are required to configure each arm. One is the tilt angle, which is the vertical angle of the arm in respect to the flat operative bed (0°) and can be adjusted by lifting upwards or downwards the arm’s nose. The other is the docking angle, which is the clockwise horizontal angle between the head of the patient (0°) and the arm’s direction. Configurations were defined by our team along with the company’s personnel (Fig. 3). Small adjustments were made during docking to optimize the angles necessary for each patient. In all operations, a bipolar fenestrated grasper was used for the left surgeon’s hand and a monopolar curved sears (with protective tip cover) for the right. The surgical procedure resembled that of laparoscopic adrenalectomy and has been previously described extensively [32]. Figure 4 includes operating room pictures during the procedures. It should be noted that the platform does not have a “memory” of the docking for each procedure and has to be manually configured separately each time.

## Results

Four female and one male patient underwent robotic-assisted lateral transabdominal adrenalectomy. Patients’ characteristics, diagnosis, operative details, and post-operative course are shown in Table 1. There were no intraoperative complications or system failures. All operations were completed without additional port placement or conversion to laparoscopic or open surgery. The console times, in chronological order, were 54, 108, 55, 61 and 29 min, respectively. The length of hospital stay was 2 days in all but one case (Table 1). In the second patient, a large para-adrenal cyst was attached to the tail of the pancreas, making dissections challenging. This led to a prolonged operative time and a longer post-operative hospital stay, due to hyperamylasemia, that lasted three days. The drain fluid was negative for elevated amylase levels. After a few days of fasting, the patient was discharged in the 8th post-operative day in excellent clinical condition and with no abnormal biochemical or radiological findings (Grade I post-operative complication, according to the Clavien-Dindo Classification [33]). However, this event cannot be attributed to the use of the Hugo™ system, but rather highlights its capabilities, since no conversion was necessary.

**Table 1:** Patients’ characteristics, operative data, and post-operative course

	Patient 1	Patient 2	Patient 3	Patient 4	Patient 5
Age (years)	73	30	65	57	78
Sex (male/female)	Female	Female	Female	Male	Female
Body Mass Index (Kg/m^2^)	22,5	19,2	24,8	26,8	30
Diagnosis	Cushing’s syndrome	Para-adrenal tumour	Cushing’s syndrome	Pheochromocytoma	Cushing's syndrome
Side (left/right)	Left	Left	Right	Left	Right
Tumour size (mm)	36	90	39	62	30
Docking time (min)	8	5	6	5	5
Robotic arms used	3	3	3	3	3
Console time (min)	54	108	55	61	29
Total operative time (min)	99	153	139	119	85
Arm collision instances	3	1	0	1	0
Intra-operative complications (yes/no)	No	No	No	No	No
Post-operative complications (yes/no)	No	Yes*	No	No	No
Length of hospital stay (days)	2	8	2	2	2
Final pathology	Adenoma	Pseudocyst	Adenoma	Pheochromocytoma	Adenoma

*Slight increase in plasma amylase levels which required fasting for a few days

In the first operation, there were some instances of clashing between the robotic arms extra-abdominally. This did not lead to any noteworthy time delay or adverse events, since there is a built-in alarm system that momentarily stops the instruments until the operator unblocks them manually. To avoid such collisions between the robotic arms, the surgical team has to first ensure that the distance of the robotic trocars is at least eight centimetres. Furthermore, small adjustments in the docking and tilt angles of the arms had to be made, to provide more ample space for each arm extra-corporeally. Lastly, abrupt manoeuvres should be avoided. By applying those principles, this issue was resolved in the following patients.

## Discussion

In this study, we demonstrated the feasibility of robotic-assisted lateral transabdominal adrenalectomy and described the configurations of the Hugo™ RAS system.

Despite concerns related to economic issues, robotic-assisted adrenalectomy, performed with the Da Vinci system, has demonstrated certain advantages over laparoscopic adrenalectomy, in terms of complications and duration of stay [13, 15, 16], especially in more difficult and challenging cases, including obese patients and hyperfunctioning tumours [10, 12, 21, 26, 34]. Moreover, also the economic issue has been challenged by recent evidence, which indicate that robotic-assisted adrenalectomy is cost-effective in Health Care Systems where inpatient care reimbursement is based on Diagnosis-Related Groups (DRGs) [26].

In general, the increased cost of robotic-assisted surgery with the Da Vinci systems is mainly attributed to unit purchase and maintenance costs, elevated instrument cost, semi-disposable instruments and longer operative times [35, 36]. This was confirmed in our centre as well, where it was demonstrated that both approaches yielded a positive income for our Institution, but less so for RAA. The higher cost of RAA resulted mainly from the increased costs of medical devices in the RAA group. Indeed, the median medical device cost for unilateral LA was EUR 796 and EUR 1770 for unilateral RAA. The higher devices cost was strongly related not only with acquisition costs but also with the number of reuses allowed for each instrument [37].

To make an investment in robotic technology more sustainable, the Hugo™ RAS platform offers two novel features. Firstly, the tower may be used also in pure laparoscopy and secondly, one of the arms can be used separately, acting as a stationary assistant in conventional laparoscopic procedures, reducing trained personnel needed.

Another, yet theoretical, proposal for reducing costs, applicable to all the available robotic platforms, is implementing an alternate charging model, where the required instruments for each procedure will be purchased as part of an operation kit. The above is expected to reduce total costs of robotic operations, making them closer to laparoscopy, especially in high volume centres that might benefit from “purchasing operations in bulk”.

Nonetheless, the main benefit of introducing a new viable robotic platform in the market, remains the further diffusion of robotics in our field and the formation of a healthy competitive environment, which by itself, is assumed to propel a further reduction in costs. Thus, the possibility to introduce the Hugo™ RAS platform, in an already established program of robotic adrenalectomy, was met with particular interest in our Institution.

As it is often true with every novel technological entry in the medical field, we observed certain pros and cons of this new platform in comparison with the established Da Vinci system. As far as technical matters are concerned, the Hugo™ has certain distinct differences from its competition, resulting mainly from its novel design. One of the main differences are the four separate arms. Each arm has a dedicated cart and can move independently. So, if the surgical team wishes to perform an operation with three arms, the fourth can be left out, saving vital space. The separate arms design also allows a great variety of modifications during setup. By adjusting the carts’ position, docking and tilt angles of each arm separately, the surgical team was able to better match the patient’s body type, operation characteristics and surgeon’s preferences. A concern that early adapters should note is the length of the robotic arms of the Hugo™ system, which predispose for collisions between the robotic arms or the arms and the assistants. The settings provided here address that issue and facilitate access of the assistants to the operating table. After overcoming this hurdle, the mobility and individual settings of each arm, allow optimization of docking for each patient. The separate arms also render the whole system more portable, allowing it to be moved with greater ease between operating rooms. This might be especially beneficial in centres that do not have a dedicated robotic operating room. In addition, each arm has six different joints, providing a wider manoeuvre range.

Contrary to the Da Vinci console, the open console of Hugo™ enabled the surgeon to sit in an upright position, while also keeping in touch with his surroundings. This allowed the surgeon to oversee the operative room and have a direct communication with the surgical team. In addition, multiple observers were able to “share” the same screen as the main operator, provided they wore the specially designed glasses, whereas in the closed Da Vinci console this was only possible via a second console, when available. The two arm-controllers with handgrips, simulating a “pistol grip”, gave a sense of familiarity to the laparoscopically experienced surgeon, potentially making easy the transition from laparoscopy. Lastly, the footswitch panel includes separate pedals for controlling the camera, energy sources, and the reserve arm, facilitating the operator’s control.

In general, the transition to this new platform was rather seamless, after proper training of the surgical team. Although this was our initial experience, docking and operative times were comparable to previous adrenalectomies performed with the Da Vinci™ platform in our centre, or reported in the bibliography [15, 16].

Of note, the Hugo™ RAS system does not have a “memory” of the docking for each procedure and must be manually configured separately each time. That makes an important difference, and probably a main limitation, with respect to the Da Vinci Xi system, which has an automated laser-guided system to facilitate docking. In the Da Vinci Xi system the projection of a green target is used to align the cart’s overhead boom to the endoscope port. Following endoscope insertion in the camera trocar and by pointing the green target towards the desired anatomical point, corresponding to the site of dissection, the system then automatically positions its boom in an optimised configuration to perform the planned procedure, thus facilitating the whole docking process.

One other notable disadvantage of the Hugo™ system is that, at its current state, it does not support fluorescent angiography with indocyanine green. Fluorescent angiography in patients requiring partial adrenalectomy facilitates precise dissection of adrenal lesions and evaluates the vasculature and viability of the remaining tissue [38]. This method is routinely applied in our centre and thus, candidates for partial adrenalectomy were excluded.

However, the above constitute our impressions from our preliminary experience with the Hugo™ platform. As we continue to utilize the new platform in parallel to the Da Vinci system, we believe that the advantages and disadvantages of each system will become more apparent in the future.

A limitation of this report is that only two of the available instruments were tested, the bipolar fenestrated grasper and the monopolar curved sears (with protective tip cover) which were functioning efficiently. In total, another seven instruments have been developed for the Hugo™ system, namely, the bipolar Maryland forceps, a large and an extra-large needle driver, a secure and a non-secure Cadiere forceps, a toothed grasper, and a double fenestrated grasper.

As any innovative robotic technology in surgery, the cost-effectiveness of this new platform remains in question. The manufacturing company claims lower total costs in capital and consumables, in comparison with the established competitor, but this has not been verified in clinical practice yet. As a result, it is not possible to draw any definitive conclusions for those matters until a sizeable number of operations are performed.

## Conclusions

The Hugo™ Robotic Assisted Surgery system is a promising platform with appealing features for the surgical community. In this initial experience, with the configurations described, we demonstrated its feasibility in performing lateral transabdominal adrenalectomy. Nonetheless, larger series and application in a wider range of procedures are needed to draw definitive conclusions.
